# Electrospinning of Poly (Acrylamide), Poly (Acrylic Acid) and Poly (Vinyl Alcohol) Nanofibers: Characterization and Optimization Study on the Effect of Different Parameters on Mean Diameter Using Taguchi Design of Experiment Method

**DOI:** 10.3390/ma15175876

**Published:** 2022-08-25

**Authors:** Tannaz Soltanolzakerin Sorkhabi, Mehrab Fallahi Samberan, Krzysztof Adam Ostrowski, Paulina Zajdel, Agata Stempkowska, Tomasz Gawenda

**Affiliations:** 1 Department of Chemical Engineering, Ahar Branch, Islamic Azad University, Ahar P.O. Box 5451116714, Iran; 2 Faculty of Civil Engineering, Cracow University of Technology, 24 Warszawska Str., 31-155 Cracow, Poland; 3 Department of Environmental Engineering, Faculty of Civil Engineering and Resource Management, AGH University of Science and Technology, Mickiewicza 30 Av., 30-059 Cracow, Poland

**Keywords:** electrospun nanofibers, electrospinning, Taguchi method, polyacrylamide, poly (acrylic acid), poly (vinyl alcohol)

## Abstract

In this study, nanofibers of poly (acrylic acid) (PAAc), polyacrylamide (PAAm) and poly (vinyl alcohol) (PVOH) were prepared using the electrospinning technique. Based on the Taguchi DOE (design of experiment) method, the effects of electrospinning parameters, i.e., needle tip to collector distance, polymer solution concentration, applied voltage, polymer solution feed rate and polymer type, on the diameter and morphology of polymer nanofibers were evaluated. Analyses of the experiments for the diameters of the polymer nanofibers showed that the type of polymer was the most significant factor. The optimal combination to obtain the smallest diameters with minimum deviations for electrospun polymer nanofibers was also determined. For this purpose, the appropriate factor levels were determined as follows: polymer PAAm, applied voltage 10 kV, delivery rate 0.1 mL/h, needle tip to collector distance 10 cm, and polymer solution concentration 8%, to obtain the thinnest nanofibers. This combination was further validated by conducting a confirmation experiment, and the average diameter of the polymer nanofibers was found to be close to the optimal conditions estimated by the Taguchi DOE method.

## 1. Introduction

Electrospinning has received increasing attention in the scientific community and industry over the past two decades, and is now seen as a critical research and commercial initiative with global economic benefits [1]. Nanocatalysis [2], tissue scaffolds [3], protective clothing [4], optical electronics [5], filtration [6], composites [7], energy storage [8] and sound absorption [9] are some of the applications of electrospun fiber mats. The electrospinning process is relatively simple. Before reaching an electrically connected electrically conductive collector, the jet solution evaporates or solidifies, and is collected as an interconnected network of filaments. The mutual repulsion of charges and the contraction of surface charges of the opposing electrode result in a force opposite to the surface tension. When the applied electrostatic forces overcome the surface tension of the fluid, the electrified fluid forms a jet from the tip of the capillary toward the grounded collection screen. At high speeds of up to thousands of revolutions per minute, the electrospun nanofibers can be oriented circumferentially. In summary, the process consists of three steps:

- jet initiation and elongation along a straight line;
- growth of the whip instability and further elongation of the jet, which may or may not be associated with branching of the jet;
- solidification of the jet into nanofibers [10,11].

These nanofibers offer various benefits, including high surface area to volume ratio, surface functionality, and exceptional mechanical properties such as tensile strength and stiffness [12]. The principle of electrospinning is based on the fact that a drop of polymer solution is placed in a strong electric field. In 1969, Taylor looked at the form of the droplet. Due to the streams ejected from the vortices, the droplet forms a cone. This cone was later named the “Taylor cone”. The effect of polymer solution feeding rate, electric field strength, and experimental conditions on the stability of nanofibers was also studied [13]. Compared to conventional spinning technologies, electrospinning produces fiber at a relatively low speed. Electrospinning produces yarn at a rate of 30 m/min, whereas conventional spinning produces yarn at a rate of 200–1500 m/min. As a result, only a few companies were interested in electrospinning as a fiber manufacturing technology until 1990 [14]. Finally, this process was defined as the formation of micro- to nanofibers from polymer solutions at atmospheric pressure and ambient temperature under the influence of a high electric field [15]. Generally, an electrospinning device consists of three main components, such as power supply (high voltage), a spinneret and a collector. As a result of the high voltage, electrical charges build up on the surface of the polymer solution. These charges repel each other to the extent that they can overcome the surface tension of the viscos solution of polymer, and form a Taylor cone in a critical electric field. A charged jet is ejected from the top of the Taylor cone and expands further in the electric field. Due to solvent evaporation, the jet eventually turns into a solid nanofiber [16]. Solution parameters, process parameters, and ambient parameters are the three types of parameters that affect the spinning process. The fiber shape is affected by the parameters in each category. As a result, these parameters can be adjusted to produce electrospun fibers with the required morphology and diameter. The most important polymer solution parameters for electrospinning are solvent used, polymer concentration, average molecular weight of the polymer, viscosity of the solution, surface tension, and electrical conductivity. In terms of the process, applied voltage, solution feeding rate, distance between collector and needle tip and spinner shape are important. Environmental parameters, for example humidity, temperature, pressure and type of atmosphere, also have an important influence [17,18,19].

Different polymers have been tested in electrospinning, such as polyvinylpyrrolidone (PVP) [20], poly (lactic acid) (PLA) [21], polyacrylonitrile (PAN) [22], poly (ethylene oxide) (PEO) [23], poly (acrylic acid) (PAAc) [24], polyacrylamide (PAAm) [25], and poly (vinyl alcohol) (PVOH) [26], and the results are promising. Most commonly, the electrospinning method is used to produce threads from PAAm, which is a water-soluble linear polymeric material derived from acrylamide monomer. PAAm is widely used in many industries including water purification, paper manufacturing, bioseparation and drug delivery, due to its hydrophilicity, excellent biocompatibility, non-specific adsorption and ease of functionalization [25,27]. The second most widely used PVOH polymer is a polyhydroxy polymer, which has been extensively studied due to its excellent film-forming and physical properties, as well as its high hydrophilicity, processability, biocompatibility and chemical resistance [26,28]. PAAc is a polymer of acrylic acid (also known as propenoic acid), which has a carboxylic group on each monomer unit. The presence of carboxylic acid groups is ionisable, which can aid in increasing the ionic strength and sensitivity to pH. This polymer becomes a polyelectrolyte in water via the dissociation of the acid groups. This polymer and poly (sodium acrylate) are thus some of the most abundantly used water-soluble anionic polyelectrolytes, e.g., as a dispersing agent, a superabsorbent polymer, an ion-exchange resin, etc. Furthermore, due to their low toxicity, they are used as a food additive [29].

The Taguchi method is a useful engineering approach for selecting optimal levels of processing parameters with minimal sensitivity to various causes of variability. Moreover, this method can also explain the influence of a large and complex number of factors on an individual and interactive basis. In general, two basic tools are required, namely, the orthogonal array (OA) to simultaneously account for several experimental design factors, and the signal-to-noise (S/N) ratio to measure the most resistant set of operational conditions to variations in performance. The Taguchi method is very appropriate for the study of nanofiber production, because the properties of nanofibers are affected by various parameters that can be optimized effectively by the Taguchi method with less labor, time and cost [30,31,32,33,34]. The number of experiments in Taguchi designs is far lower than full factorial designs, which makes them an attractive alternative to factorial designs. For example, for five factors each at three levels, the total number of experiments in a full factorial design would be 3^5^ = 243. However, using a Taguchi design of experiment, the effects of five factors at three levels can be investigated with only 27 experiments. In the literature, the Taguchi method has been used to optimize electrospinning parameters of various polymer nanofibers [30,35,36,37,38,39,40,41,42,43]. The Taguchi methodology uses standard tables, termed as orthogonal arrays, to arrange the combination parameter values in an experimental design. It employs three different options for target design; i.e., “bigger is better”, “smaller is better” and “nominal is better”. The choice of these targets depends on the type of process and product. For our case, we have chosen “smaller is better” to get the best designs with the minimum nanofiber diameters.

In the present study, polymer nanofibers were fabricated from poly (acrylic acid) (PAAc), polyacrylamide (PAAm) and poly (vinyl alcohol) (PVOH) polymers by the electrospinning method. The Taguchi DOE method was used to determine the optimal combination of parameters, such as needle-to-collector distance, polymer solution concentration, applied voltage, polymer solution delivery rate, and polymer type, to minimize the diameter size and its variation in the polymer nanofibers.

## 2. Materials and Methods

### 2.1. Polymers

**Scheme 1:**
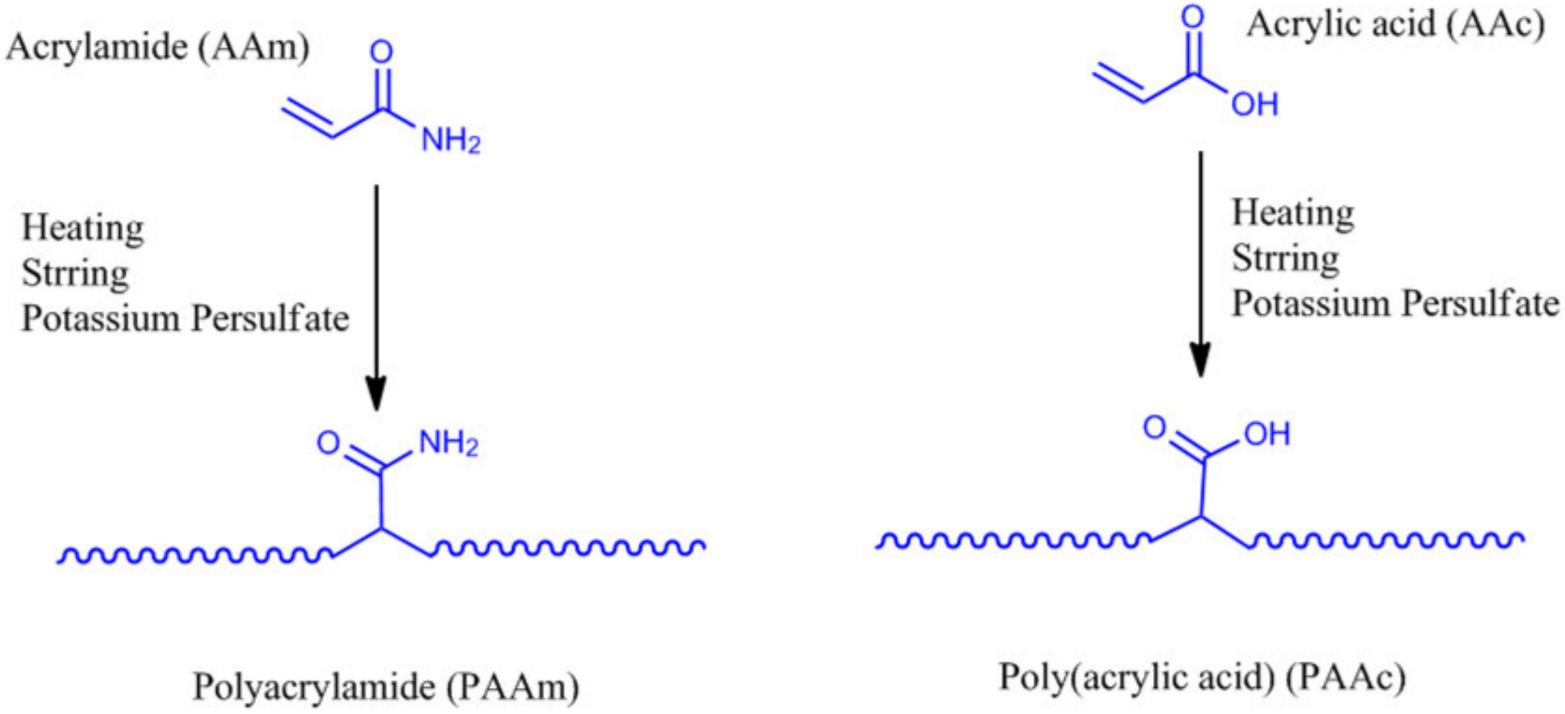
Reaction scheme of free radical polymerization, and PAAm and PAAc preparation.

Acrylamide (C_3_H_5_NO, AAm, Merck, Germany, purity: ≥99%), acrylic acid (C_3_H_4_O_2_, AA, Merck, Germany, purity: ≥99%), poly (vinyl alcohol) (PVOH) with a molecular weight of 72,000 g/mol (Merck, Germany), potassium persulfate (K_2_S_2_O_8_, KPS, Panreac Química, Spain, purity: ≥98%) and distilled water were used. PAAc and PAAm were prepared by free radical polymerization in aqueous solution. The reactions were carried out in the presence of KPS as an initiator (2% in monomers) at 62 ± 2 °C under nitrogen atmosphere for 2 h. Water was then removed from the samples under reduced pressure and the samples were dried in an oven at 70 °C for 12 h to obtain solid PAAc and PAAm. Scheme 1 shows the reaction scheme of synthesis of the these polymers. Polymer solutions were prepared in distilled water at different concentrations by stirring the mixture magnetically for 4 h until homogeneous solutions were obtained. Three different concentrations, i.e., 8, 10 and 14 wt. % PVOH, PAAc and PAAM, were used in the present study.

### 2.2. Electrospinning

**Figure 1:**
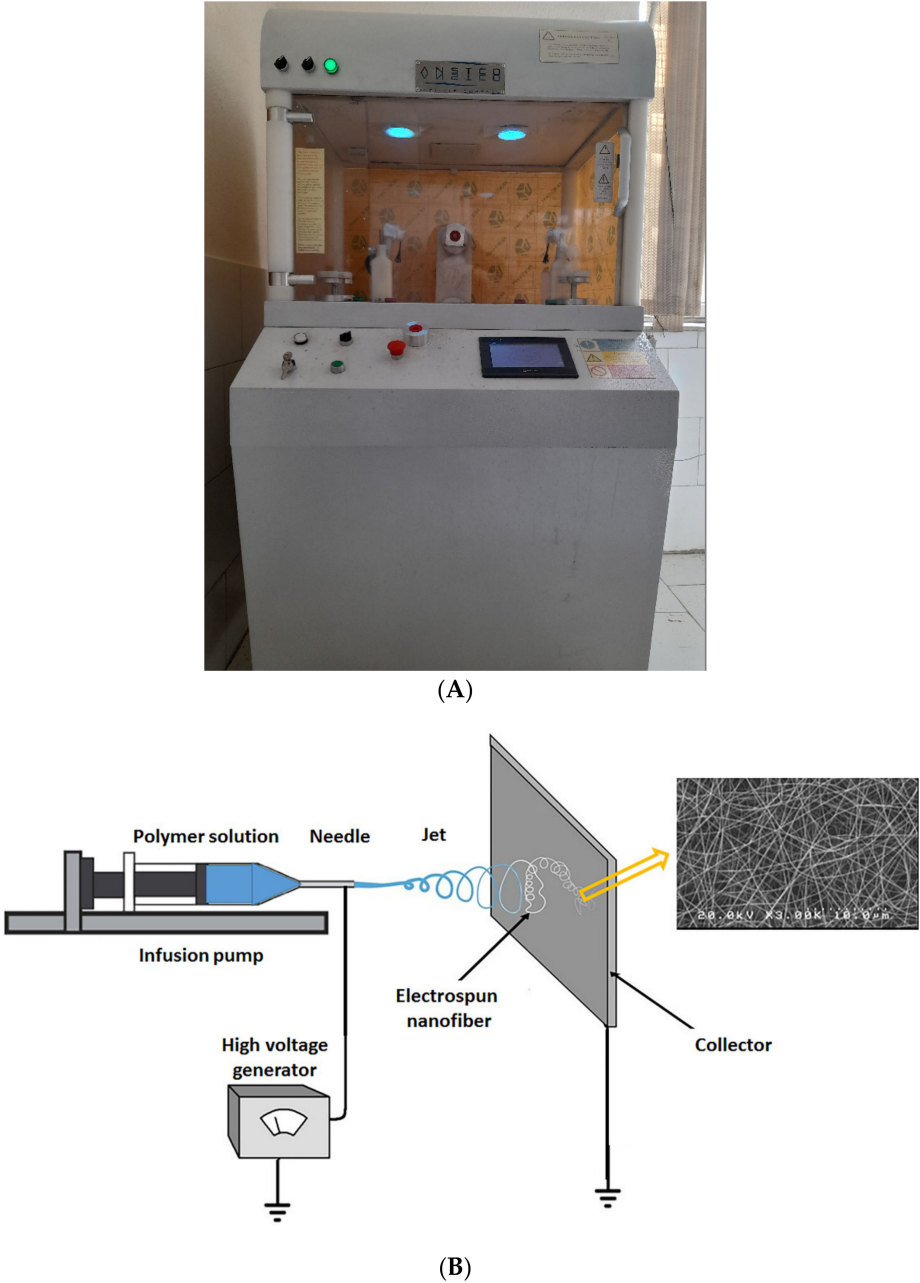
The electrospinning machine (**A**) and the schematic of electrospinning setup (**B**).

The solutions of PAAc, PAAm, and PVOH were converted into nanofibers by electrospinning. The electrospinning apparatus (ANSTCO-N/ VI, Asian Nanostructures Technology Company (ANSTCO), Tehran, Iran) used in this study and the schematic of the electrospinning setup are shown in Figure 1. The electrospinning setup consisted of a high-voltage power supply, a plastic syringe, a needle, and a collection plate. The flow rate was controlled by a pump connected to the plastic syringe. During the electrospinning process, most of the solvent evaporates rapidly and charged fibers remain, which can be collected on a plate (collector). The selected parameters and their levels used in the current study for the electrospinning process are given in Table 1.

To provide conductive surfaces, nanofibers gathered on a collector (aluminum foil) by electrospinning were sputtered with a thin layer of gold. The morphology and size of the nofibers from PAAc, PAAm, and PVOH were observed and analyzed using a field emission scanning electron microscope (FESEM, Hitachi model S-4160, Japan-Daypetronic Company, Tokyo, Japan). After FESEM analysis, FESEM micrographs were also taken from different locations using Image J open source image analysis software to determine the size and average diameter of nanofibers obtained by electrospun. Measurements in the image analysis software were performed for different locations of each FESEM micrograph, so that the number of individual nanofibers evaluated in each case was approximately 20.

The production of PAAc, PAAm and PVOH nanofibers was achieved by implementing the Taguchi method using Minitab 18 statistical software. An L 27 design was chosen using a factorial design of five parameters with three levels. A total of twenty-seven experimental runs was planned and performed. Table 2 shows the L27 orthogonal array with the independent factors P, C, D, V and F, which represent “the type of polymer”, “polymer solution concentration” (wt. %), “the distance between the collector and the needle” (cm), “applied voltage” (kV), and “the solution feeding rate” (mL/h), respectively, as well as the response, which indicates the average diameter of the resulting nanofiber.

### 2.3. Design and Analysis of Experimental Parameters by the Taguchi Method

Analysis of variance (ANOVA) was used to determine the parameters that significantly affect the quality of the polymer nanofibers, and to obtain the optimal diameter and electrospinning conditions. Taguchi’s optimal design of experiments is the key to the successful application of ANOVA or other statistical analyses. The total variation (ST), sum of squares for each of the five factors (Si), and percentages (%) were calculated by ANOVA. The following equation expresses the total variation (ST), which is the sum of the squares of all trial results:

(1) $$S_{T}=\left[\overset{N}{\underset{i=1}{\sum }}{\bar{Y}}_{i}^{2}\right]-\left[\frac{{\left(\sum_{i=1}^{N}{\bar{Y}}_{i}\right)}^{2}}{N}\right]$$

where ${\bar{Y}}_{i}$ is the mean fiber diameter and N is the number of trials in the Taguchi DOE study. Five factors of electrospinning, such as the type of polymer, polymer solution concentration, the distance between the collector and the needle, applied voltage, and the solution feeding rate at three different levels, were mentioned as the sum squares S_P_, S_C_, S_D_, S_V_ and S_F_, respectively. CF stays constant for all factors, and is the correction factor. All sums of squares are calculated by the correction factor (CF).

(2) $$S_{P}=\frac{P_{\mathrm{PAAc}}^{2}}{3}+\frac{P_{\mathrm{PAAm}}^{2}}{3}+\frac{P_{\mathrm{PVOH}}^{2}}{3}-\mathrm{CF}$$

(3) $$S_{C}=\frac{C_{8}^{2}}{3}+\frac{C_{10}^{2}}{3}+\frac{C_{12}^{2}}{3}-\mathrm{CF}$$

(4) $$S_{D}=\frac{D_{10}^{2}}{3}+\frac{D_{14}^{2}}{3}+\frac{D_{18}^{2}}{3}-\mathrm{CF}$$

(5) $$S_{V}=\frac{V_{12}^{2}}{3}+\frac{V_{16}^{2}}{3}+\frac{V_{20}^{2}}{3}-\mathrm{CF}$$

(6) $$S_{F}=\frac{F_{0.1}^{2}}{3}+\frac{F_{0.2}^{2}}{3}+\frac{F_{0.3}^{2}}{3}-\mathrm{CF}$$

The percentage contribution of the five factors (PP, PC, PD, PV and PF) is the ratio of the total variance of each factor (S_P_, S_C_, S_D_, S_V_ and S_F_) to total variation (ST), as given by:

(7) $$P_{i}=\frac{S_{i}}{S_{T}}\times 100$$

where i is the number of factors (i = 5 for this study).

Optimum combination factors identified by using a “smaller the better” characteristic formula to minimize the nanofiber diameter and its variation in the electrospinning process, as given below:

(8) $$\frac{S}{N}=-10\;\log\left(\frac{1}{n}\overset{n}{\underset{i=1}{\sum }}y_{i}^{2}\right)$$

where S/N is the signal-to-noise ratio, y is the diameter of electrospun polymer nanofibers and n is the number of measurements. Mathematically, the greater the value of signal-to-noise ratio, the smaller the variance of the diameter.

## 3. Results and Discussion

### 3.1. Nanofiber Morphology and Diameter

**Figure 2:**
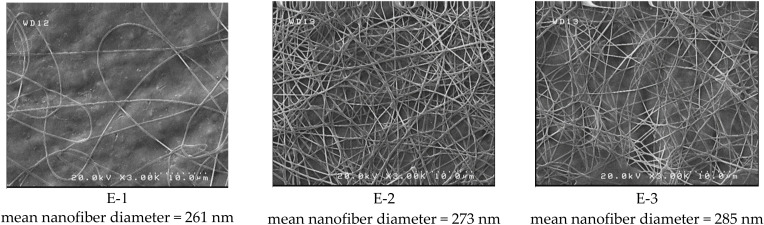

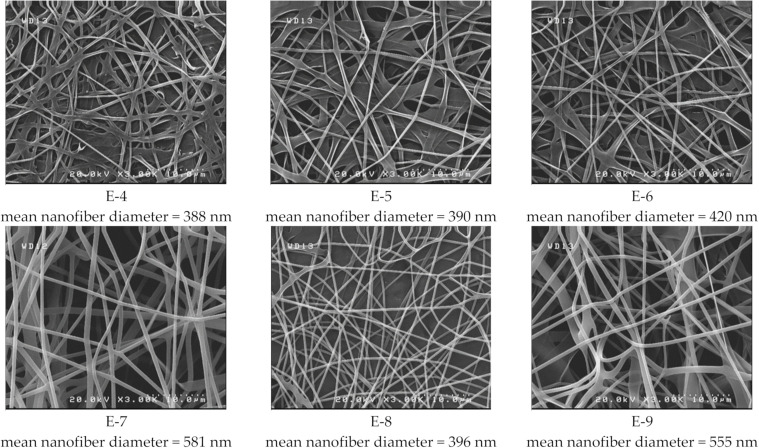
FEEM images of PAAc nanofibers related to experiments 1–9 of the Taguchi method.

**Figure 3:**
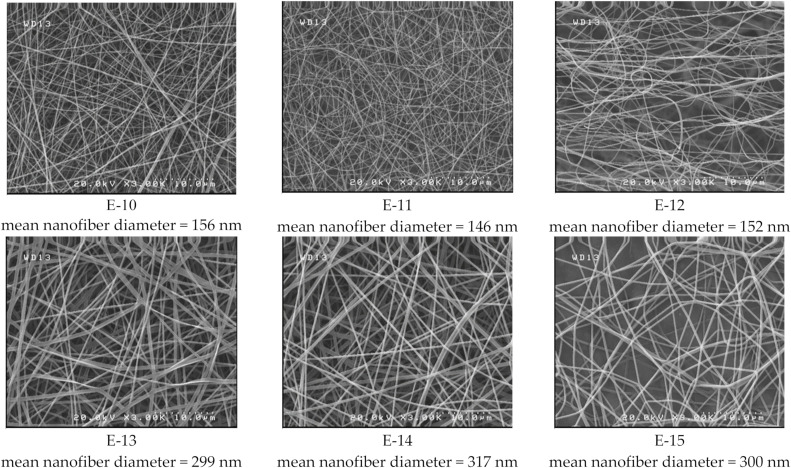

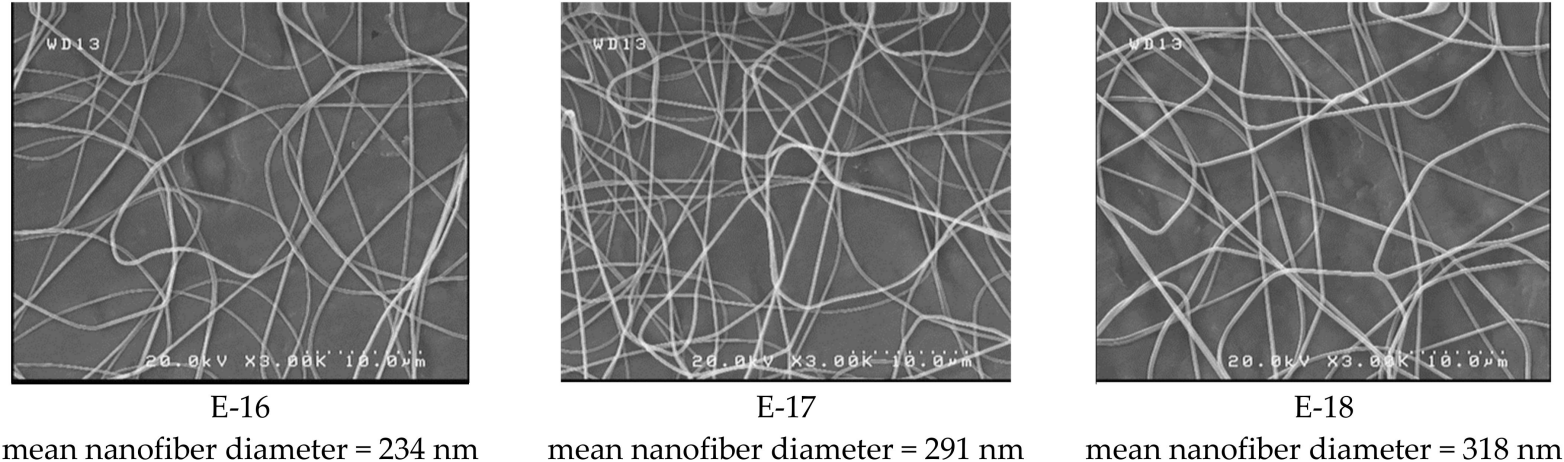
FEEM images of PAAm nanofibers related to experiments 10–18 of the Taguchi method.

**Figure 4:**
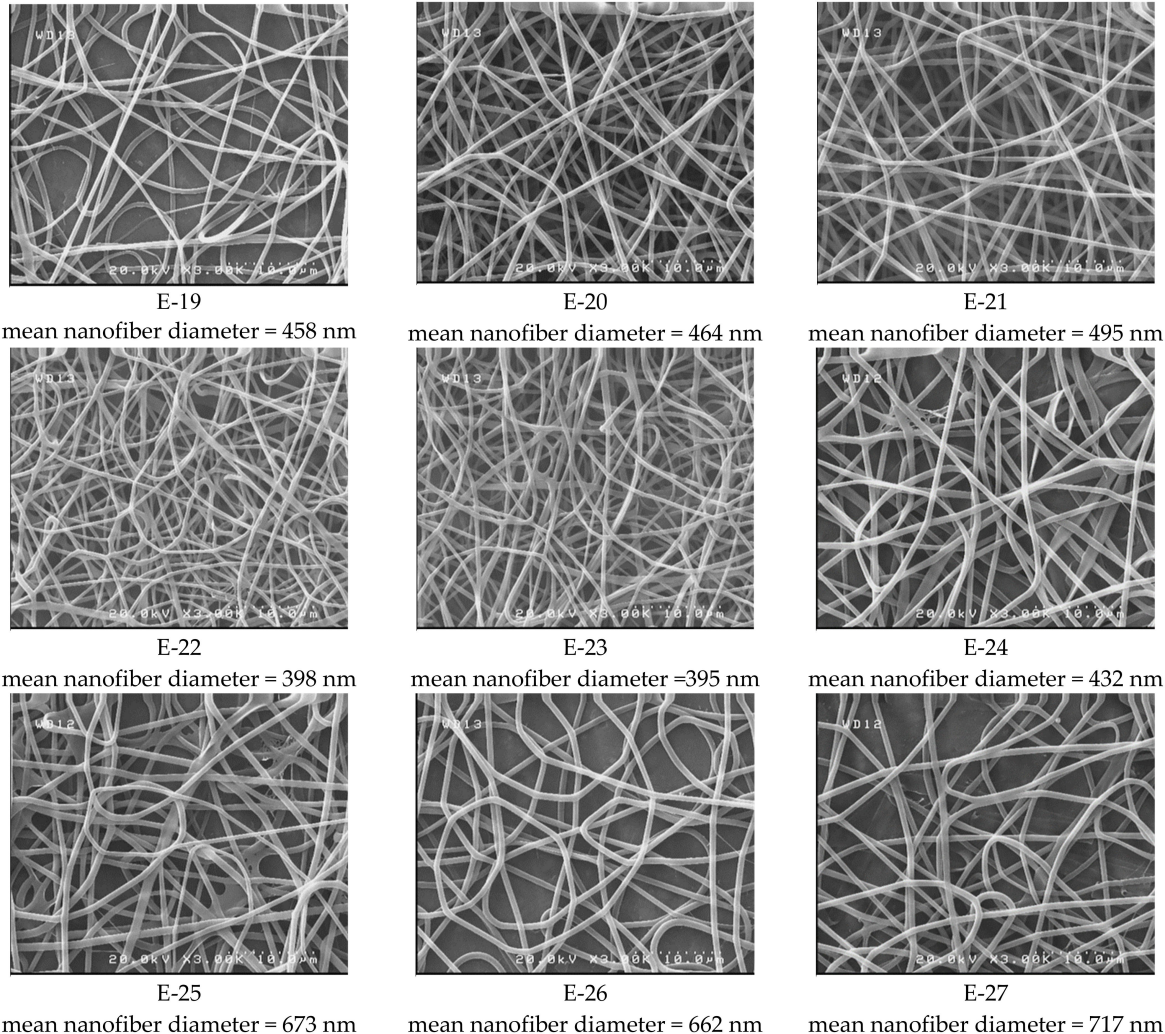
FEEM images of PVOH nanofibers related to experiments 19–27 of the Taguchi method.

Figure 2, Figure 3 and Figure 4 show the FESEM micrographs and surface morphologies of electrospun polymer nanofibers produced by electrospinning. From the FESEM results, it was found that the electrospinning process formed polymer nanofibers with smooth surfaces, oriented randomly on the collection plate.

**Figure 5:**
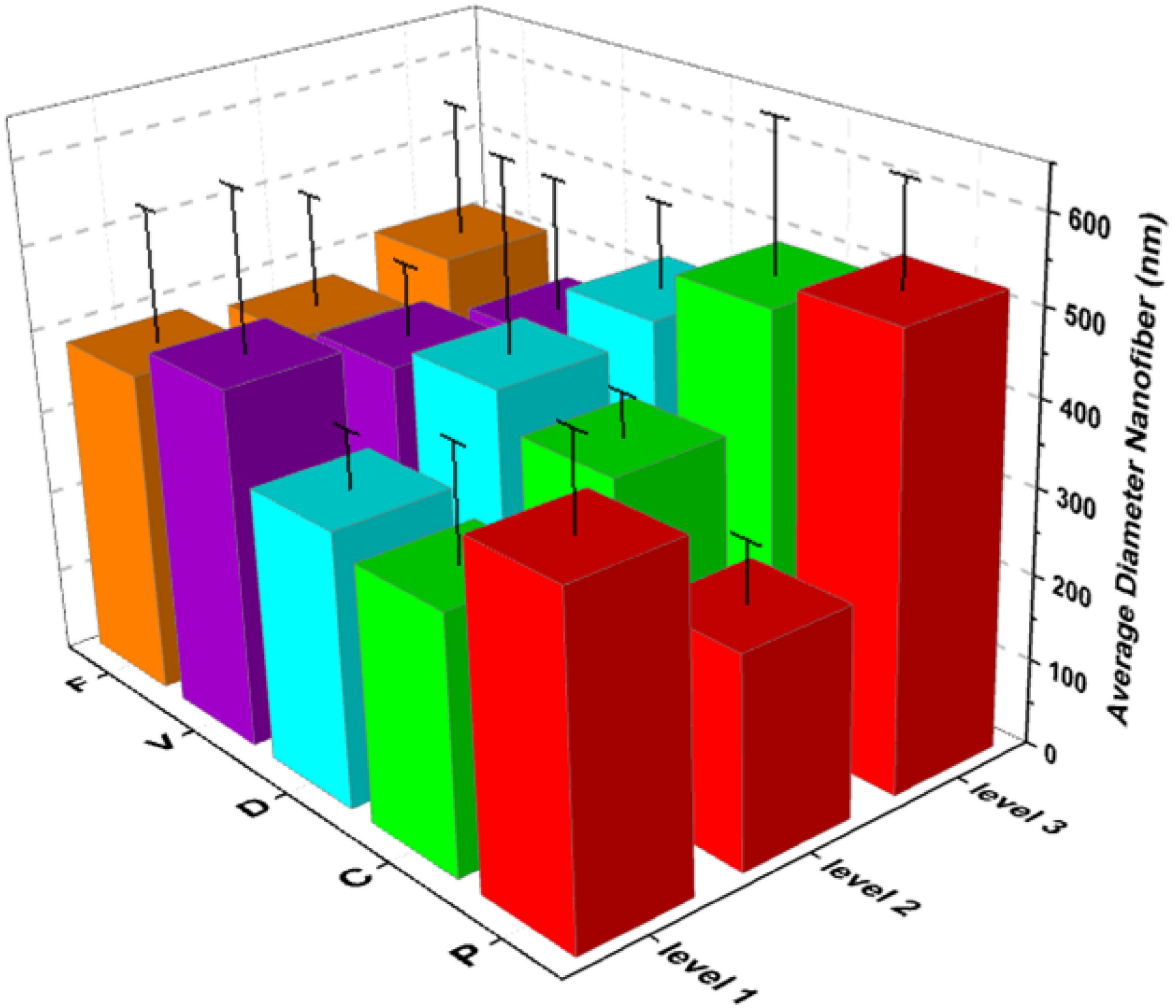
The effect of distance between the needle and collection plate (D), the concentration of polymer solution (C), the applied voltage (V), the solution feeding rate (F) and type of polymers (P) on the average electrospun nanofiber diameter.

The diameters and standard deviations of the obtained nanofibers are shown in Table 2. E-27 produced the largest average nanofiber diameter (717 ± 78 nm). This PVAOH nanofiber was produced at level 3 concentration (12), level 2 distance (14), level 1 applied voltage (12), and level 3 feed rate (0.3). The type of polymer, the highest concentration of polymer solution and the lowest level of applied voltage were the main reasons for the increase in the average diameters of nanofibers. However, the average diameters of polymer nanofibers decreased drastically at E-10, E-11 and E-12, where the average diameters and standard deviations were 156 ± 18 nm, 146 ± 15 nm and 152 ± 12 nm, respectively. The polymer type, i.e., PAAm, was one of the reasons for the decrease in mean diameters in these three series. Other reasons for this were electrospinning at the lowest concentration and highest applied voltage. Table 3 and Figure 5 show the effects of polymer type, polymer solution concentration, collector-to-needle distance, applied voltage, and solution feed rate on the average diameters of electrospun nanofibers.

The diameter of the electrospun polymer nanofibers was found to decrease with increasing applied voltage. When a voltage higher than the critical voltage is applied, charged jets are ejected from the Taylor cone. In fact, when an electric field is applied to a droplet of polymer solution at the tip of needle, the surface of the droplet becomes charged and the electric force overcomes the surface tension force; as a result, the electrically charged jet is formed. A higher voltage increases the electrostatic repulsion force on the charged jet, resulting in a decrease in the diameter of the electrospun polymer nanofibers [44]. The results also show that the concentration of polymer solution in the electrospinning process plays an important role in determining the polymer nanofiber diameter. The diameter decreases as the concentration decreases. This can be attributed to the fact that when the polymer content in the electrospinning solution (concentration of polymer chains) decreases, the viscosity of the solution decreases, resulting in less entanglement of the polymer chains in the solution, which leads to the formation of nanofibers with a smaller diameter [45]. It can also be seen in Figure 5 that decreasing the distance between the electrodes causes the diameter of nanofibers to decrease. The difference in collection distance (distance between needle tip and collector) has a direct effect on the electric field strength. When the distance between the collector and the needle is smaller, the electric field strength increases. As a result, the applied force and elongation increase in the electrospinning process, and the diameter of the electrospun polymer nanofibers decreases.

In Figure 5, it can be seen that the effect of the solution feeding rate as another parameter in the electrospinning process is complex. This is because, with an increased solution feeding rate, a larger volume of polymer solution is pulled away from the needle tip. However, when the flow rate is insufficient to achieve the fiber extraction rate, the formation of continuous nanofibers and the electrospinning process may be interrupted. Under this condition, the Taylor cone at the needle tip disappeared.

### 3.2. Analysis of Variance (ANOVA)

**Figure 6:**
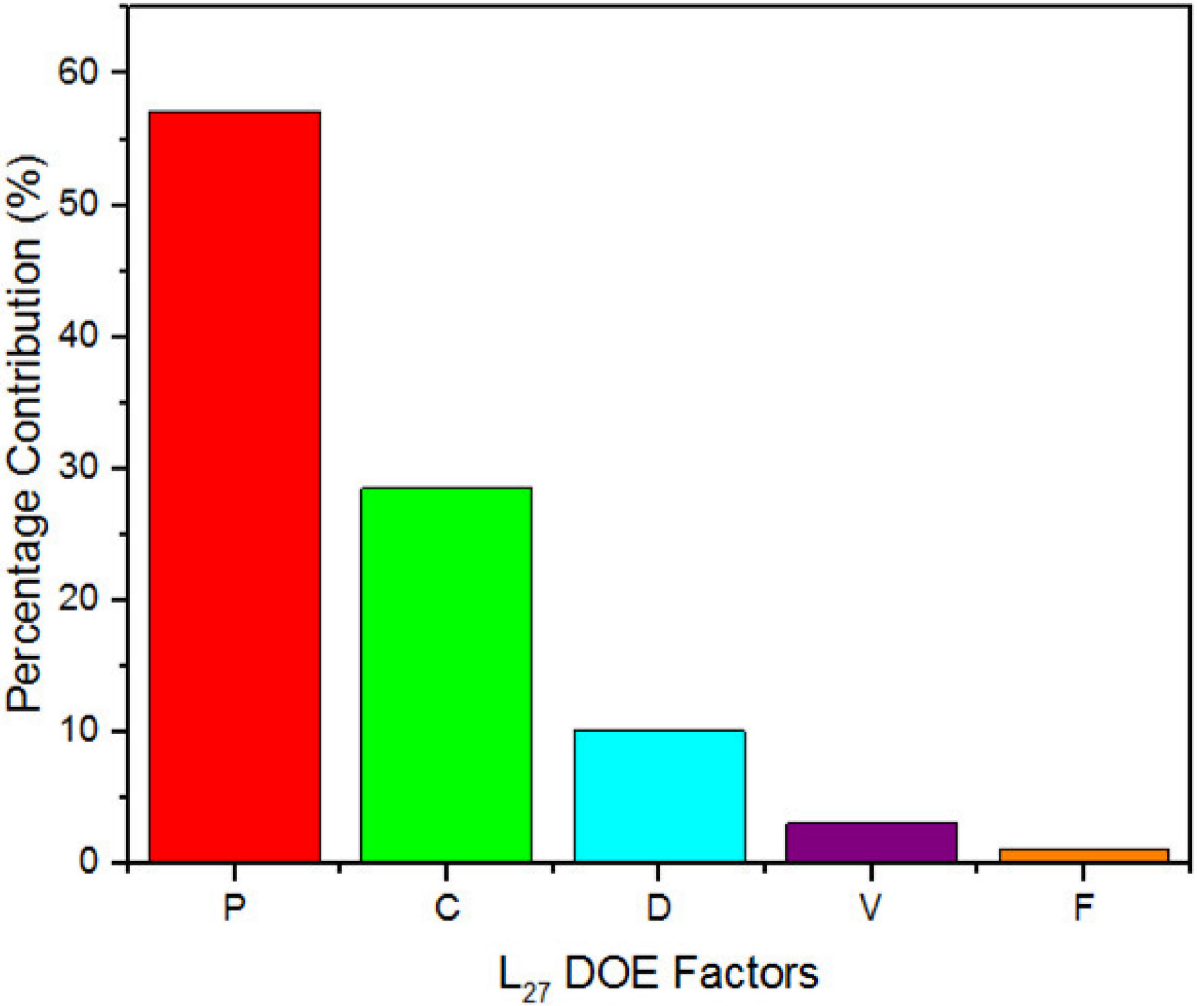
ANOVA diagram for the determination of significant factors influencing electrospun polymer nanofiber diameters.

The relative percentage contributions of the factors of the electrospinning process were determined using Equations (1)–(6), and the results are shown in Figure 6. The ANOVA diagram shown in Figure 6 indicates the effects of electrospinning parameters on electrospun polymer nanofiber diameters by the percentage contributions for selected L27 DOE factors. Polymer type (P factor) was a significant variable determining the diameter of polymer nanofibers, with a percentage of 57.17%. This shows that the electrospinning of the PAAm solution greatly reduces the diameter of polymer nanofibers. The polymer solution concentration (factor C) and the distance between the collector and the needle (factor D) were ranked second and third in terms of the influence on reducing the diameter of the polymer nanofibers, with percentages of 28.50% and 10.10%, respectively. The applied voltage (factor V) and solution delivery rate (factor F) were found to be insignificant, and were only 3.09% and 1.12%. This shows that the applied voltage and solution feed rate (factor F) have minimal effects on the diameters of nanofibers, which can be maintained within a feasible experimental range.

### 3.3. Optimum Combination of Factors

It is important to understand that the S/N ratio is the most important decision parameter in the Taguchi optimization method for analyzing experimental data. In this work, the S/N ratio must be the maximum in order to obtain the optimum conditions for nanofiber production according to the Taguchi optimization technique. Table 2 shows the experimental results in terms of nanofiber diameters and S/N ratios calculated according to the principle of “the smaller, the better”. The best candidate for inclusion in the optimal combination was E-11, which achieved a maximum S/N value of 43.28, with a mean nanofiber diameter and standard deviation of 146 ± 15 nm. Another strong candidate is E-12, which has an S/N value of 43.63, with a mean nanofiber diameter and standard deviation of 152 ± 12 nm. The mean fiber diameter of E-11 is slightly smaller than that of E-12.

**Figure 7:**
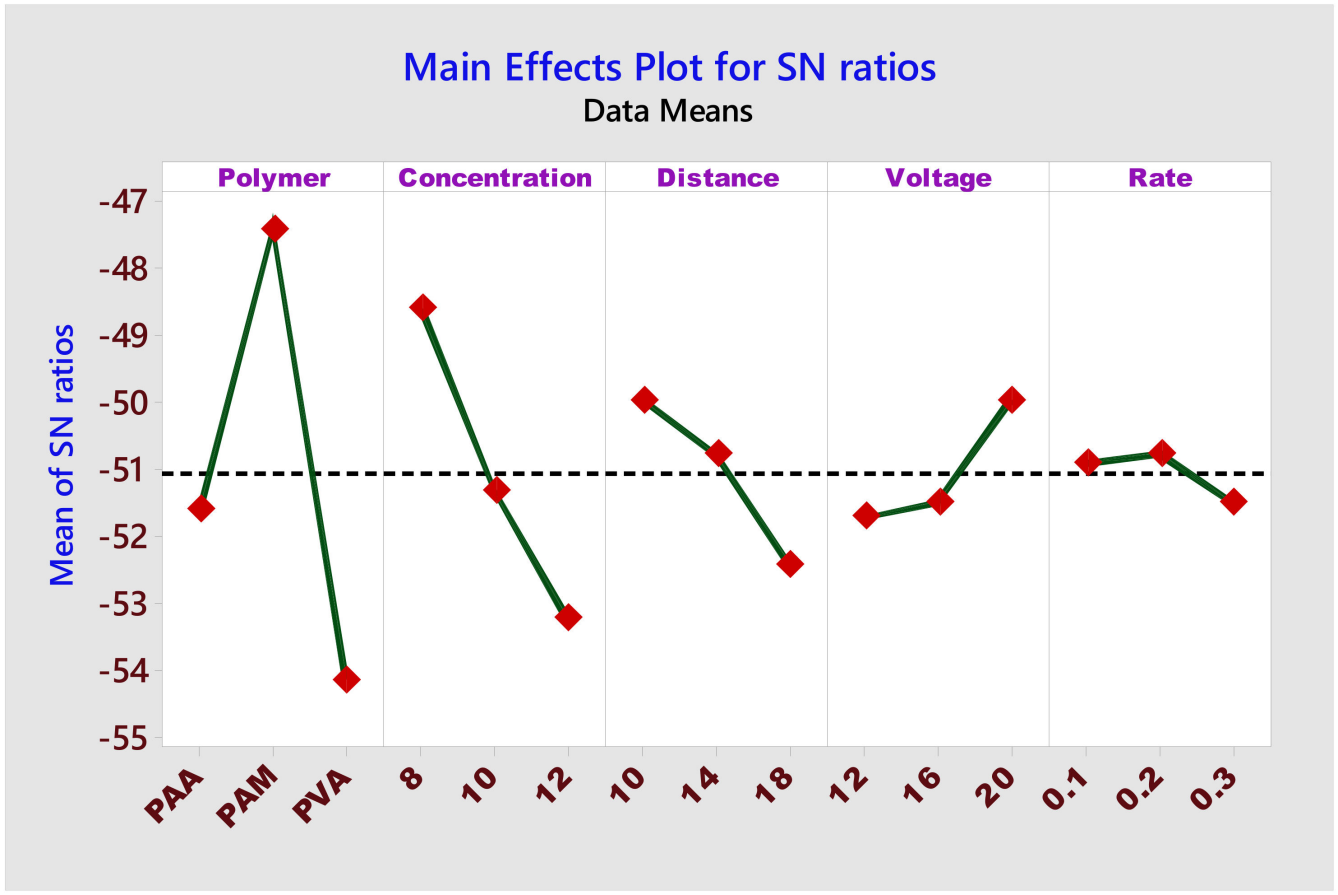
Average S/N ratio diagram for the determination of the optimum combination of factors for electrospun polymer nanofibers with the smallest nanofiber diameter size and its minimum variation.

The average S/N ratio for each parameter level is shown in Figure 7. It shows that the optimal conditions for the fabrication of polymer nanofibers by electrospinning are PAAm polymer, polymer solution concentration of 8 wt. % (C1), an applied voltage of 20 kV (V3), a needle tip-to-collector distance of 10 cm (D1), and a feed rate of 0.2 mL/h (F2). It can also be seen that the type of polymer is the most significant parameter affecting the diameters of nanofibers. The polymer concentration appeared to be the second most important parameter, while the distance of the needle tip from the collector, the solution feeding rate and the applied voltage appear to be relatively unimportant.

### 3.4. Confirmation Experiment to Optimum Conditions

**Figure 8:**
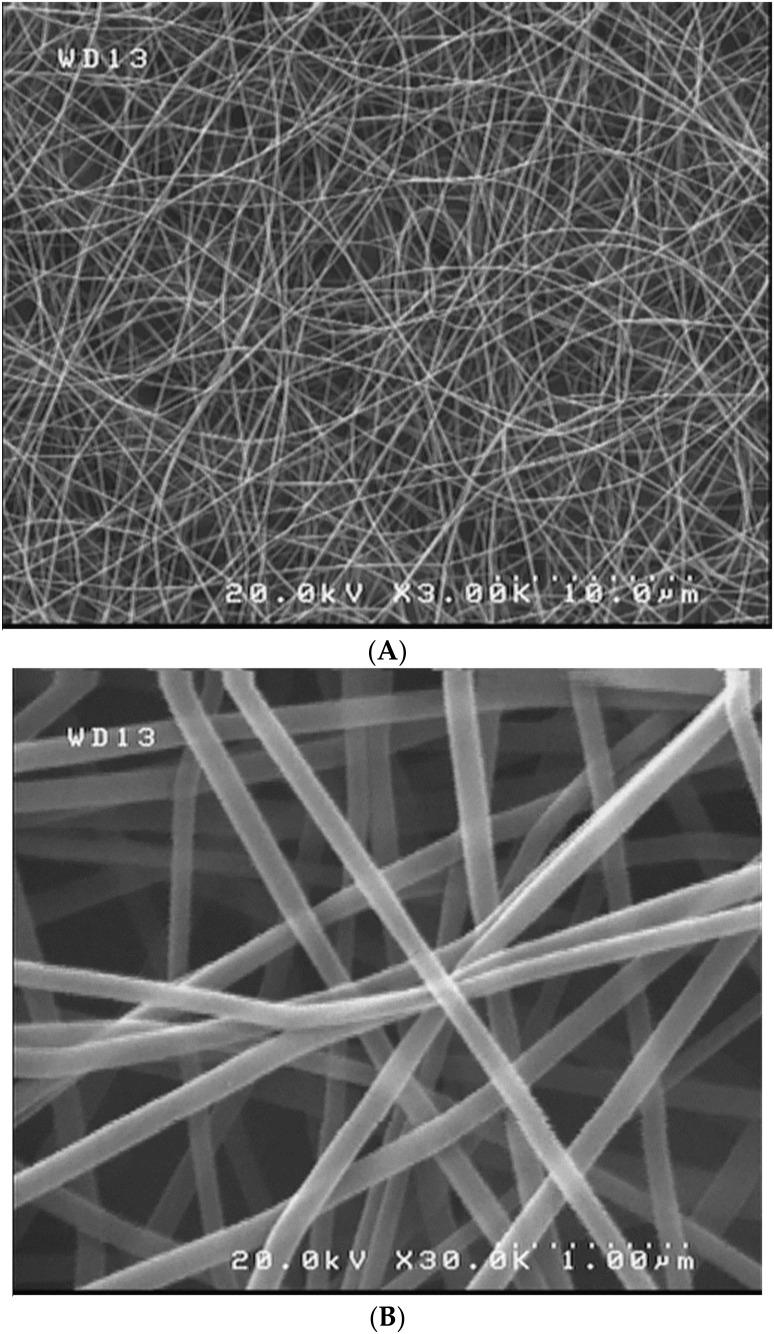
FESEM micrographs of the confirmation experiment with magnifications of 3000 (**A**) and 30,000 (**B**) for the optimum combination of factors.

The experiment to confirm the optimal combination of factors was conducted as a necessary and important step of the Taguchi DOE method. In order to investigate the effects on the morphology and diameter of electrospun polymer nanofibers, the optimal combination was tested under the same conditions as before. Figure 8 shows typical FESEM images from the confirmation experiment. The randomly oriented electrospun PAAm nanofibers had smooth surfaces, without defects in the images. The average nanofiber diameter and standard deviation were 148 ± 10 nm for the confirmation experiment. The diameter of these nanofibers was compared with the best Taguchi candidate values obtained in experiments E-11 (146 ± 15) and T8 (152 ± 12). Experiment E-11 yielded the smallest nanofiber diameter, but the optimal combination yields relatively small-diameter polymer electrospinning nanofibers, with the smallest standard deviation.

## 4. Conclusions

An L27 orthogonal array along with signal-to-noise (S/N) ratios and ANOVA in the Taguchi DOE method were used to investigate the needle tip-to-collector distance, the concentration of polymer solution, the applied voltage, the feeding rate of the polymer solution, and the type of polymer at three different levels on the electrospun polymer nanofiber diameter. The small nanofiber diameter with the minimum variance has been found to be controlled mainly by two significant factors, namely, type of polymer and polymer solution concentration. The optimum combination of factors with the highest level of the applied voltage, and the lowest levels of polymer solution concentration and needle tip-to-collector distance, was found along with a feeding rate of 0.2 mL/h and PAAm polymer solution. It was also determined that the type of polymer and the polymer concentration are the two most significant parameters in the electrospinning process, and needle tip-to-collector distance, polymer solution feeding rate and the applied voltage appear to be relatively insignificant.

## Figures and Tables

**Table 1:** Experimental parameters and their levels.

Parameter	Abbreviation	Units	Level 1	Level 2	Level 3
Type of polymer	P	-------	PAAc	PAAm	PVOH
Polymer solution concentration	C	% (*w*/*v*)	8	10	12
Distance	D	cm	10	14	18
Applied voltage	V	kV	12	16	20
Solution feeding rate	F	mL/h	0.1	0.2	0.3

**Table 2:** Experimental design with mean nanofiber diameter (nm), standard deviation (SD) and signal-to-noise ratio (S/N).

	Parameters					Fiber Diameter		S/N Ratio
Exp. No.	P	C	D	V	F	Mean (nm)	SD	
E-1	PAAc	8	10	12	0.1	261	60	−48.33
E-2	PAAc	8	10	12	0.2	273	55	−48.72
E-3	PAAc	8	10	12	0.3	285	51	−49.09
E-4	PAAc	10	14	16	0.1	388	103	−51.77
E-5	PAAc	10	14	16	0.2	390	76	−51.82
E-6	PAAc	10	14	16	0.3	420	79	−52.46
E-7	PAAc	12	18	20	0.1	581	85	−55.28
E-8	PAAc	12	18	20	0.2	396	139	−51.95
E-9	PAAc	12	18	20	0.3	555	88	−54.88
E-10	PAAm	8	10	12	0.1	156	18	−43.86
E-11	PAAm	8	10	12	0.2	146	15	−43.28
E-12	PAAm	8	10	12	0.3	152	12	−43.63
E-13	PAAm	10	14	16	0.1	299	22	−49.51
E-14	PAAm	10	14	16	0.2	317	42	−50.02
E-15	PAAm	10	14	16	0.3	300	20	−49.54
E-16	PAAm	12	18	20	0.1	234	38	−47.38
E-17	PAAm	12	18	20	0.2	291	7	−49.27
E-18	PAAm	12	18	20	0.3	318	39	−50.04
E-19	PVOH	8	10	12	0.1	458	73	−53.21
E-20	PVOH	8	10	12	0.2	464	82	−53.33
E-21	PVOH	8	10	12	0.3	495	65	−53.89
E-22	PVOH	10	14	16	0.1	398	8	−51.99
E-23	PVOH	10	14	16	0.2	395	73	−51.93
E-24	PVOH	10	14	16	0.3	432	59	−52.71
E-25	PVOH	12	18	20	0.1	673	147	−56.56
E-26	PVOH	12	18	20	0.2	662	53	−56.41
E-27	PVOH	12	18	20	0.3	717	78	−57.11

**Table 3:** Means of nanofiber diameters (nm) and standard deviation (SD).

	Means of Nanofiber Diameters			Standard Deviation		
Level	1	2	3	1	2	3
P	394	246	522	108	71	119
C	299	371	492	133	49	173
D	321	412	429	67	218	101
V	421	384	357	187	82	159
F	383	370	408	158	135	158

## Data Availability

The data presented in this article are available within the article.
